# Plasma Proteomic Profiling in HIV-1 Infected Methamphetamine Abusers

**DOI:** 10.1371/journal.pone.0031031

**Published:** 2012-02-16

**Authors:** Gwenael Pottiez, Teena Jagadish, Fang Yu, Scott Letendre, Ronald Ellis, Nichole A. Duarte, Igor Grant, Howard E. Gendelman, Howard S. Fox, Pawel Ciborowski

**Affiliations:** 1 Department of Pharmacology and Experimental Neuroscience, Omaha, Nebraska, United States of America; 2 University of Nebraska Medical Center, Omaha, Nebraska, United States of America; 3 University of California San Diego, San Diego, California, United States of America; George Mason University, United States of America

## Abstract

We wanted to determine whether methamphetamine use affects a subset of plasma proteins in HIV-infected persons. Plasma samples from two visits were identified for subjects from four groups: HIV+, ongoing, persistent METH use; HIV+, short-term METH abstinent; HIV+, long term METH abstinence; HIV negative, no history of METH use. Among 390 proteins identified, 28 showed significant changes in expression in the HIV+/persistent METH+ group over the two visits, which were not attributable to HIV itself. These proteins were involved in complement, coagulation pathways and oxidative stress. Continuous METH use is an unstable condition, altering levels of a number of plasma proteins.

## Introduction

Methamphetamine (METH) is an widely abused drug worldwide [1], not only because of its general harmful effects on organ function and physiology, but also because its use can lead to the transmission of HIV and other infectious diseases. This commonly occurs through needle sharing and risky sexual behaviors [2], [3]. The use of METH itself has adverse effects on the central nervous system (CNS) with a broad range of clinical outcomes [4]. The acute effects of METH depend on the amount of drug used and route of administration [5]. With longer term use, METH is neurotoxic, damaging dopaminergic nerve terminals, most profoundly in the striatum [6], [7]. Long-term dopamine depletion and microglial activation is implicated in METH-induced neurotoxicity through reactive oxygen species, increased release of 5-hydroxytryptamine (5-HT), and loss of function in tryptophan hydroxylase and the serotonin transporter [7]. METH also has untoward actions on the immune system, yielding predominantly inhibitor effects [8], [9], [10]. Due to its effects on the immune and the central nervous systems, the multiple effects of METH are further complicated in the context of HIV-1 infection. When combined, METH use and HIV infection may have even more devastating effects [2], [11], [12], [13].

Mechanisms underlying cognitive impairment resulting from HIV-1 infection and concurrent METH use are far from understood and diagnosis is currently limited to clinical evaluation and neurocognitive assessments [14]. During the last decade much experimental effort has been directed towards discovery of biomarkers in body fluids to aid in diagnosis and monitoring of neurocognitive disease, with a special emphasis on blood which is the easiest clinical sample to obtain. Protein biomarkers are an especially important target, and blood contains useful and unique proteomic signatures – proteins and their fragments potentially changed due to pathological changes. However there are several obstacles in progress of discovery new biomarkers [15] such as linking particular biomarkers to pathology of disease and weakness of quantitation, therefore proper validation techniques of individual proteins is a very complex procedure [16]. We aimed to obtain clues to the effects of METH use on the blood plasma proteome. To accomplish this, our study was designed to capture changes in host responses following different durations of METH use or abstinence in HIV-infected individuals. We compared changes between two visits for in four groups of individuals. The first three groups consisted of HIV-infected METH users who either continued abusing METH (HIV+/persistent METH+), subjects who stopped METH and reported a short period (e.g., less than 12 weeks) of abstinence (HIV+/ST METH abstinent), or subjects who stopped METH and reported a longer period (e.g., at least 12 weeks) of abstinence (HIV+/LT METH abstinent). A fourth group consisted of uninfected controls who did not meet criteria for a METH use disorder and who reported no use of METH (HIV−/METH−). Among numerous methods used in proteomic profiling, we selected an isobaric tag for relative and absolute quantitation (iTRAQ) shotgun proteomic approach. This tandem mass spectrometry (MS/MS) profiling platform enables the quantification of identified peptides which is further translated to protein quantitation. Such large-scale acquisition and analysis can generate statistically significant valuable information with the ability to address existing questions and formulate new hypotheses, as successfully implemented here.

Profiling of samples from HIV+ individuals who report abstinence from METH may allow for a better understanding of the molecular mechanisms underlying reversible (short-term), and potentially non-reversible (long-term) effects, of METH use within the context of HIV-1.

## Materials and Methods

### Clinical samples

The University of California San Diego (UCSD) and University of Nebraska Medical Center Institutional Review Boards (IRB) approved the studies in which samples were collected, and the UCSD and UNMC IRBs approved their use in this study. Written informed consent was received from all participants. To ascertain whether subjects might have decisional impairment, a Decisional Capacity Assessment was administered. This consisted of an 11-item post-consent quiz with questions regarding the nature of the illness being studied, the voluntary nature of participation, and the ability to withdraw at any time, the consequences of withdrawing, the possible risks and benefits of participation, the procedures involved, the time required, confidentiality, and whom to call with any questions. Assistance with reading or understanding the vocabulary was provided. Inability to achieve a perfect score on the test or other serious indication of questionable capacity resulted in further evaluation by a clinical psychiatrist, psychologist, or neurologist. All subjects in this analysis achieved a perfect score on the test, indicating satisfactory demonstration of capacity.

All participants were ambulatory and underwent evaluation in the outpatient research center at UCSD. Eligibility criteria included the ability to undergo a structured clinical interview and to provide details of combined antiretroviral therapy (cART) use and substance use history.

Data were collected according to a standardized protocol of comprehensive neuromedical, neurobehavioral, and laboratory assessments as described previously [17]. Briefly, the following clinical parameters were evaluated using structured interviews and laboratory assessments where appropriate: cART use, including current and past exposure and HIV disease markers (plasma viral load and current and nadir CD4+ cell counts). Blood was collected by venipuncture and used to quantify plasma HIV viral loads by a commercial reverse transcription polymerase chain reaction assay (nominal lower quantitation limit, 50 copies/mL [Amplicor; Roche Diagnostic Systems, Indianapolis, Indiana]). Current CD4+ cell counts were measured by flow cytometry. Psychiatric diagnoses, including substance use disorders (abuse and dependence), were assessed using the computer assisted Composite International Diagnostic Interview (CIDI) [18], [19], a fully structured clinical interview that is widely used in psychiatric research [20], [21]. The CIDI classified current (within in the last 30 days) and lifetime (>30 days ago) histories of METH use (e.g., abuse and dependence) disorders.

### Materials

Ammonium phosphate, α-cyano-4-hydroxycinammic acid (CHCA), trifluoroacetic acid (TFA) were from Sigma Aldrich (St. Louis, MO, USA). HPLC grade water and acetonitrile (ACN) were from Fisher Scientific (Pittsburg, PA, USA). NuPAGE 4–12% gels were from Invitrogen Corp. (Carlsbad, CA, USA). Ready Gel™ Blotting Sandwiches and glycine were from Bio-Rad (Hercules, CA, USA). Super Signal® West Pico chemiluminescent substrate was from Pierce (Rockford, IL, USA). Mouse monoclonal antibodies against vitamin D-binding protein (Abcam, Cambridge, MA, USA), and ceruloplasmin (BD Biosciences, San Jose, CA, USA) were used as primaries, for detection of secondary antibodies were polyclonal goat HRP-conjugated anti-mouse IgG (Jackson ImmunoResearch, West Grove, PA, USA).

### Sample processing

Samples had been stored at −80°C from the time of collection until the time of shipment. Samples were shipped on dry ice from UCSD to UNMC and, on arrival, remained frozen. HIV was inactivated in all samples by addition of 10 µl of 10% Triton-X100 freshly made and 50 µl of cocktail of protease inhibitors (Sigma-Aldrich St. Louis, MO) per 1 mL of sample. After 30 minutes samples were aliquoted and those unused were stored at −80°C. Two hundred fifty microliters from each sample were filtered using 0.2 µm spin filter and immunodepleted using an IgY14 column (Sigma-Aldrich) connected to a liquid chromatography systems HPLC to immunodeplete abundant plasma proteins: albumin, α_1_-antitrypsin, IgM, haptoglobin, fibrinogen, α_1_-acid glycoprotein, apolipoprotein A-I and A-III, Apolipoprotein B, IgG, IgA, transferrin, α_2_-macroglobulin and complement C3. Flow-through fractions containing unbound proteins were concentrated using Vivaspin 15R (Sartorius, Aubagne, France), according to the manufacturer protocol. Finally, protein concentration was determined with a NanoDrop spectrophotometer (Thermo Scientific, San Jose, CA).

### Trypsin digest and peptide labeling

Fifty micrograms of proteins were precipitated with ethanol. Briefly, we added 10 volumes of cold ethanol (200 proof) to each sample. Samples were incubated for 3 h at −20°C and centrifuged at 13000× *g* for 15 minutes at 4°C. Proteins pellets were washed with 1 ml of 70% ethanol and dried in SpeedVac (ThermoScientific). Subsequent solutions were provided with iTRAQ reagent kit (Applied Biosystem (ABI), Carlsbad, CA).

Dried proteins were solubilized with dissolution solution and proteins were denatured with 1 µl of denaturant reagent. Proteins reduction with reducing reagent was performed for 1 h at 60°C and finally cysteine blocking solution was used to block cysteines during 10 minutes at room temperature.

Trypsin from ABI was reconstituted at 1 µg/µl with milliQ water and 10 µg of trypsin were added to each sample. Digestion was performed for 16 h at 37°C.After digestion, peptides were labeled with iTRAQ label reagent (ABI) at room temperature for 2 h and 8 samples, labeled with the different isobaric mass tags, were combined in one tube and dried with SpeedVac.

### iTRAQ labeled peptides processing

Having subsequent samples collected from the same individuals at various times allowed us to make comparisons between time points as well as across all groups. Because we had four groups and two time points per group, the 8-plex iTRAQ approach to quantify changes was the method of choice. Table 1 shows how samples were “scrambled” into four sets to remove tag labeling bias. Since Group 1, (HIV+/persistent METH+) had only four individuals, four samples were randomly chosen from those available in Groups 2, 3 and 4 (consisting of 8, 12 and 10 identified subjects, respectively). One set of samples utilized all eight iTRAQ tags and each was measured in a separate analytical run.

**Table 1:** iTRAQ tag assignation for 4 biological replicates in experimental design.

Sample set	Tag 113	Tag 114	Tag 115	Tag 116	Tag 117	Tag 118	Tag 119	Tag 121
**1**	*P1, G2, V1	P2, G4, V2	P2, G4, V1	P3, G1, V2	P1, G2, V2	P4, G3,V1	P4, G3, V2	P3, G1, V1
**2**	P5, G2, V2	P6, G3, V1	P7, G1, V1	P6, G3, V2	P8, G4, V1	P7, G1,V2	P5, G2, V1	P8, G4, V2
**3**	P9, G3, V2	P10, G1, V1	P11, G2, V2	P9, G3, V1	P11, G2, V1	P12, G4,V2	P10, G1, V2	P12, G4, V1
**4**	P13, G4, V2	P14, G2, V1	P15, G1, V2	P13, G4, V1	P16, G3, V1	P14, G2,V2	P15, G1, V1	P16, G3, V2

*P = Patient; G = Group; V = Visit.

Samples were clarified using mix education exchange (MCX) column (Water Corp., Milford, MA). Label peptides were solubilized with 1 ml of 0.1% formic acid, passed through the column, then the column was first washed with 5% methanol, 0.1% formic acid solution and then with methanol HPLC grade. Peptides were eluted with 1.4% NH_4_OH in methanol [22].

Samples were dried, reconstituted in 1.44 ml of 0.1% formic acid and supplemented with 1.44 µl of OFFGEL solution for 360 µl of peptides. Next, samples were fractionated based on their isolelectric point (*pI*) using 3100 OFFGEL Fractionator (Agilent, Inc. Santa Clara, CA). OFFGEL strips were rehydrated for 15 minutes at room temperature with 40 µl of OFFGEL solution. Peptide samples were loaded onto strips, splitting them equally between all 12 wells. Separation was performed for 20,000 Vhrs.

Collected fractions were cleaned with C-18 spin columns, according to the manufacturer's protocol. Briefly, fractions were adjusted to 5% acetonitrile (ACN) and 0.5% trifluoric acid (TFA) and passed through activated columns. Columns were washed twice with a 5% ACN, 0.5% TFA solution and peptides were eluted with a 70% ACN, 0.1% TFA solution. Peptides were finally dried and stored at −80°C until further use.

### Off line LC-MS/MS analysis

Subsequent fractionation of OFFGEL fractions was performed off-line using Tempo™ LC system with automatic high density spotting onto MALDI target plates. Peptides were solubilized in 20 µl of 0.1% TFA and 10 µl of samples were loaded onto a ProteoCol™ C18 trap cartridge (MichromBiosources, Auburn, CA) and washed for 20 minutes at 9 µl/min. Gradient of separation was realized using a ratio between two buffers, Water∶ACN∶TFA (98∶2∶0.1) (Buffer A) and water∶ACN∶TFA (2∶98∶0.1) (Buffer B). To perform the separation, the subsequent gradient was applied, time 0 to 5 min 5 to 15% buffer B, 5–52 minutes 15–35%, 52–54 minutes 35–80%, 54–64 minutes 80%, 64–65 minutes 80—5% and 65–72 minutes 5%. Peptide elution was monitored with a UV cell at 214 nm absorbance. After the UV cell, eluted peptides were mixed with a matrix solution (1.2 mg/ml in 75% ACN and 0.1% TFA solution) at a flow rate 1 µl/minute using a Harvard Apparatus syringe pump. Fractions were spotted every 30 seconds and voltage applied to the plate during spotting was 2.8 kV.

Spotted fractions were submitted for data acquisition on a 4800 MALDI-TOF/TOF mass spectrometer (ABI). MS spectra were acquires from 800 to 3000 *m/z*, for a total of 1000 laser shots by an Nd-YAG laser operating at 355 nm and 200 Hz. Laser intensity remain fixed for all the analyses. MS/MS analyses were performed using 1 kV collision energy with air as CID gas. Metastable ions were suppressed, for a total of 1000 laser shots.

Protein identification and quantification were performed with ProteinPilot™ software using Paragon method. The search parameters were as follows: iTRAQ 8plex (peptide labeled), Methylthio alkylation of Cysteine, NCBI database restricted to *Homo sapiens*.

### Statistical analysis

Protein/peptide abundance measures generated by iTRAQ were first pre-processed using ProteinPilot™ v2.0. The logarithm of the abundance measure was modeled as a function of animal, protein, peptide, tag and experimental condition. Four sets of samples were used to simultaneously collect data from eight experimental conditions composed of two visits from four groups: Group 1: HIV+/persistent METH+, Group 2: HIV+/ST METH abstinent, Group 3: HIV+, LT METH abstinence, Group 4: HIV-/METH-. All available abundance records (N = 77,159) were normalized using an iterative back fitting procedure to remove the animal, protein and peptide effects. Comparison of the distribution of the protein/peptide normalized (log) abundance measures by experimental condition was restricted to records with ProteinPilot™ assessed “confidence” of at least 50%. The mixed model was fit on the normalized abundances of each protein to adjust for the correlation of the samples collected from the same subject. The abundances of each protein between two visits within the same group were compared, and the relative abundance and the p-values were calculated to evaluate the short/long term effects of METH within each group.

### Western blot - validation

Western blot quantitation was done as previously described [22], [23]. 1DE was performed using NuPAGE gel system (Invitrogen Corp.) in 4–12% gradient Bis-Tris gels under reducing conditions and transferred onto PVDF membrane. Chemiluminescent signal was detected using SuperSignal West Pico™Chemiluminescent Substrate and recorded on Blue Lite X-ray film (ISCBioExpress, Kaysville, UT).

## Results

### Subjects

All subjects visited the research center at least two times during the study, which allowed us to measure changes in the plasma proteome between visits. Subject demographic and clinical characteristics are summarized in Table 2. Group 1 (HIV+/persistent METH+) has relatively few subjects due to the rarity of such individuals. METH users entering the study were offered resources for treatment for their drug abuse if requested (Groups 1, 2 and 3). The study took place in an outpatient setting in which sobriety/abstinence was not required, thus effects of discontinuation of METH, rather than initiation of METH, were observed. Blood was drawn during visits and first blood sample was taken at the first visit. This measure is indirect because it attempts to infer the effects of METH from its discontinuation rather than from its administration. On the other hand, it is sufficient given ethical guidelines because ethically the direct effects of transition from a normal state to one of METH abuse cannot be clinically studied.

**Table 2:** Subject demographic, psychiatric, and clinical characteristics.

Variables	Group 1 (N = 4)		Group 2 (N = 8)		Group 3 (N = 12)		Group 4 (N = 10)	
***Demographics:***								
Age, years (mean, SD)	37.8	±8.9	38.1	±5.6	43.6	±9.0	38.4	±9.6
Sex, % male (N, %)	3	75%	8	100%	12	100%	7	70%
Education, years (mean, SD)	10	±2.3	13	±1.9	14	±2.0	12.8	±2.3
Ethnicity, % Caucasian, N, %)	2	50%	7	88.0%	10	83.0%	5	50.0%
***Psychiatric Characteristics:***								
METH Use Disorder (N, %)^a^								
Lifetime (>30 days ago)	4	100%	8	100%	12	100%	0	0%
Current (≤30 days ago)	2	50%	3	37.5%	0	0%	0	0%
Age of 1st use of METH, years (mean, SD)^b^	15.7	±4.0	24.3	±7.5	31.1	±30.0	–	–
Days since last use of METH (mean, SD)	5.1	±3.0	5.0	±2.7	1174.2	±3395.0	–	–
Total days used METH (mean, SD)	527.0	±912.9	735.7	±2080.8	1100.3	±2624.7	–	–
Total grams of METH used (mean, SD)	5569.5	±9646.7	677.3	±1915.6	477.5	±889.5	–	–
***Clinical Characteristics*** c ***:***								
On CART (N, %)	0	0%	5	63%	6	50%	–	–
Plasma VL (median, IQR)	4.3	2.4, 5.1	4.4	3.0, 5.2	1.7	1.7, 3.7	–	–
Nadir CD4 (median, IQR)	422	119, 953	181	23, 282	168	76, 385	–	–
Current CD4 (median, IQR)	579	209, 942	238	76, 427	416	323, 705	–	–

Abbreviations: CART = combination antiretroviral therapy; SD = standard deviation; VL = viral load log10 copies/mL; IQR = interquartile range.

a METH use disorder defined as meeting DSM-IV criteria for METH abuse or dependence.

b These variables are restricted to METH+ subjects.

c These variables are restricted to HIV+ subjects.

### Protein identification and quantitation

Plasma proteomic profiling is made difficult by the presence of a number of abundant proteins, making examination of less abundant proteins not feasible. Furthermore the sheer number of proteins and the large dynamic range of their concentrations further complicate analysis. Thus we used multidimensional separation techniques – immunodepletion of the 14 most abundant proteins, separation of peptides into bins by isoelectric points (pI), and then further separation of these pI-based bins by reverse phase liquid chromatography. We chose the iTRAQ method of quantitation, and then eliminating label bias by a scrambling strategy. The data from mass spectrometry analysis were analyzed separately for each sample set and the results of all samples sets were then combined, revealing the identification of a total of 390 proteins. Statistical analysis revealed that 28 proteins were significantly differentially expressed in the persistent METH users; these are listed in Table 3.

**Table 3:** Summary of differentially expressed proteins with statistical significance.

Protein names	NCBI accession number	UniProtKB accession number	pI	MW (kDa)
Alpha 2 macroglobulin variant	gi|167017130	A8K2U0	5.8	161.1
Alpha-1-antichymotrypsin	gi|1340142	P01011	5.5	49.8
Alpha-2-glycoprotein 1, zinc-binding	gi|52790422	P25311	5.7	34.3
Alpha-2-HS-glycoprotein (Fragment)	gi|7770227	P02765	6.4	22.7
Alpha2-HS glycoprotein	gi|2521983	P022765	5.4	39.4
Antithrombin III	gi|179161	P01008	6.3	52.6
Apolipoprotein B-100 precursor	gi|28780	P04114	6.6	515.6
C4A variant protein	gi|443671	P0C0L4	6.8	193.7
cDNA FLJ55673, highly similar to Complement factor B	gi|194384366	B4E1Z4	6.8	140.9
cDNA FLJ56652, highly similar to Hemopexin	gi|221044726	B7Z2Q4	6.4	15.7
Ceruloplasmin precursor	gi|119599290	P00450	5.5	116.8
Coagulation factor XI precursor	gi|4503627	P03951	8.5	70.1
Complement C5 preproprotein	gi|38016947	P01031	6.1	188.3
Fibrinogen gamma chain	gi|70906437	P02676	5.7	49.5
Group-specific component (vitamin D-binding protein)	gi|34785355	P02774	5.3	52.9
Guanine nucleotide regulatory protein	gi|404722	Q14344	8.1	44.1
Hemopexin precursor	gi|386789	P02790	6.6	51.4
Immunoglobulin kappa light chain V	gi|21669423	No UniProtKB number*	6.7	29.3
Inter-alpha globulin inhibitor H2	gi|70778918	P19823	6.4	106.5
Inter-alpha-trypsin inhibitor family heavy chain-related protein	gi|221042206	B7Z544	6.2	98.3
Kininogen-1 isoform 2	gi|156231037	P01042	6.3	72
Leucine-rich alpha-2-glycoprotein 1	gi|21707947	P02750	6.5	38.2
Plasminogenisoform 1 precursor	gi|4505881	P00747	7.0	90.6
PREDICTED: similar to immunoglobulin lambda-like polypeptide 1	gi|239752604	No UniProtKB number*	8.9	53.3
Prothrombin; coagulation factor II	gi|1335344	P00734	5.5	69.3
Putative uncharacterized protein DKFZp686G11190	gi|34365282	Q6MZQ6	8.3	52.0
Recombinant IgG3 heavy chain	gi|9857757	No UniProtKB number*	8.4	89.0
Vitamin D-binding protein/group specific component	gi|455970	P02774	5.3	52.9

*The protein sequences have no homolog sequence in UniProtKB database.

### Effect of persistent METH use and short and long term abstinence

**Figure 1:**
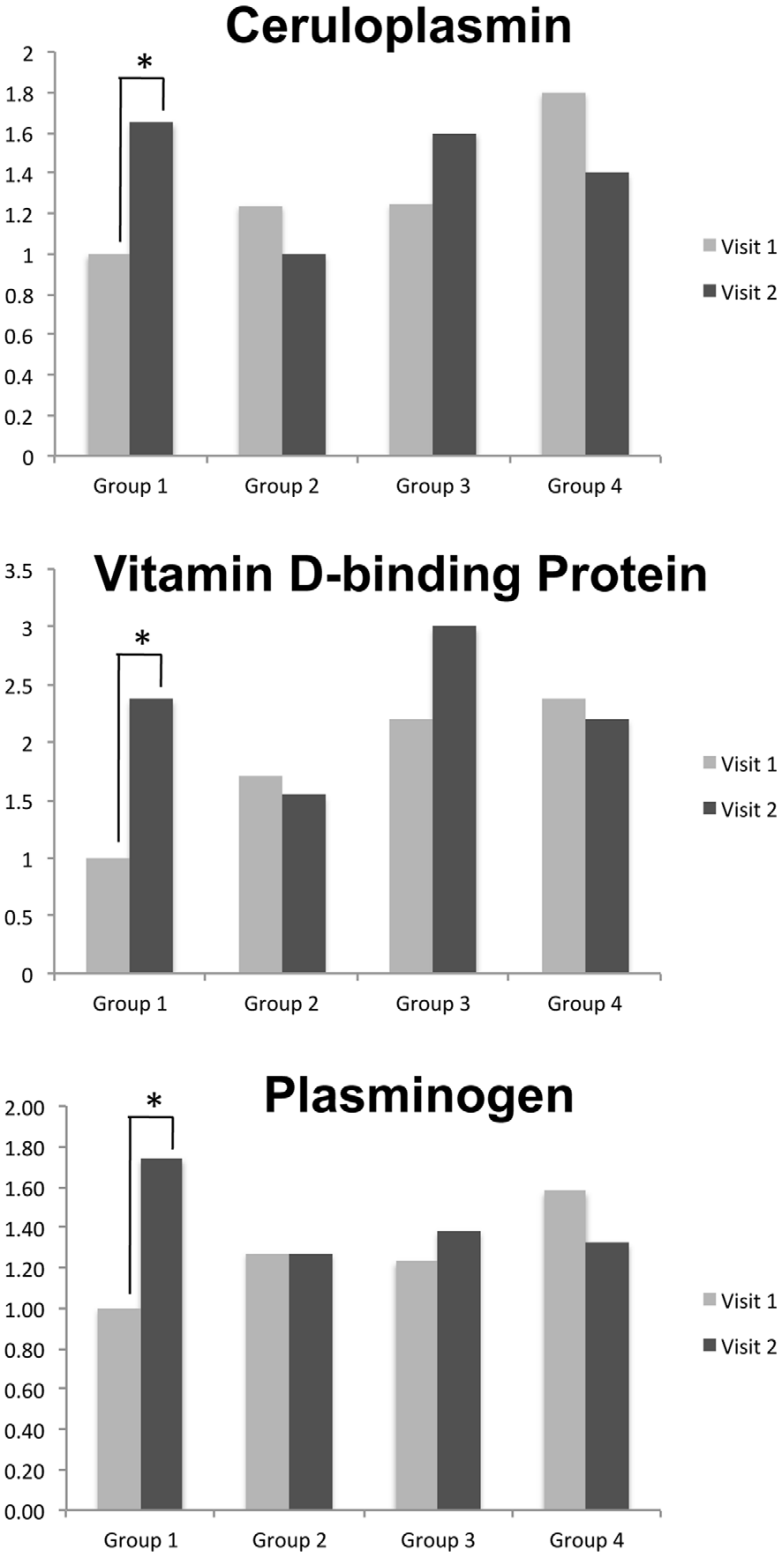
Quantitative changes in expression of ceruloplasmin, vitamin D-binding protein and plasminogen. Based on iTRAQ ratios, all three proteins showed significant increase from visit one to visit two in Group 1. No changes in other groups of subjects were found.

The primary objective of this study was to gain insights into changes in plasma proteome induced by METH in HIV-infected individuals. The study was designed based on participants who successfully stopped using drugs of abuse, therefore observed effects measured such change, rather than change induced directly by drug administration. Changes between visits in all four groups are summarized in Table 4. Group 1 measures changes in proteome in HIV-infected individuals when METH was still used, revealing a large number of changed proteins between visits. Groups 2 (i.e., reported stopping using METH over the course of the study for between two to 12 weeks) and Group 3 (i.e., reporting having at least three months of abstinence) who showed relatively few changes between visits. Similarly Group 4 (e.g., not HIV-infected and did not use METH) did not show many changes, thus in normal individuals the proteome is relatively stable as measured by our methods. Fig. 1 shows differences between visits for three selected proteins. Statistical significance (increase) was observed only in Group 1.

**Table 4:** Changes in plasma protein expression levels between visits in all groups.§

Names	Group 1	p-value	Group 2	p-value	Group 3	p-value	Group 4	p-value
Alpha 2 macroglobulin variant	1.44	<0.05	−1.10	NS	1.35	NS	1.17	NS
Alpha-1-antichymotrypsin	**2.09**	<0.001	−1.01	NS	**1.84**	<0.01	**2.01**	<0.01
Alpha-2-glycoprotein 1, zinc-binding	−1.12	NS	**−1.94**	<0.05	1.02	NS	**−1.82**	<0.05
Alpha-2-HS-glycoprotein (Fragment)	**3.86**	<0.001	−1.48	NS	1.09	NS	−1.01	NS
Alpha2-HS glycoprotein	**2.94**	<0.001	**−1.52**	NS	1.14	NS	1.12	NS
Antithrombin III	**2.25**	<0.001	1.02	NS	1.43	<0.05	1.08	NS
Apolipoprotein B-100 precursor	1.20	NS	1.41	NS	**1.60**	<0.05	1.36	NS
C4A variant protein	1.44	<0.005	1.06	NS	−1.04	NS	−1.27	NS
cDNA FLJ55673, highly similar to Complement factor B	1.33	<0.05	1.15	NS	1.44	<0.005	−1.32	NS
cDNA FLJ56652, highly similar to Hemopexin	**2.21**	<0.001	−1.13	NS	1.24	NS	−1.15	NS
Ceruloplasmin precursor	**1.65**	<0.001	−1.19	NS	1.29	NS	−1.29	NS
Coagulation factor XI precursor	**1.89**	<0.05	−1.31	NS	1.42	NS	1.01	NS
Complement C5 preproprotein	**2.01**	<0.05	1.30	NS	1.28	NS	**1.83**	NS
Fibrinogen gamma chain	−1.43	NS	−1.31	NS	**1.57**	NS	**−1.61**	NS
Group-specific component (vitamin D-binding protein)	**3.05**	<0.001	−1.13	NS	**1.53**	NS	−1.03	NS
Guanine nucleotide regulatory protein	**−2.63**	NS	1.27	NS	−1.50	NS		NS
Hemopexin precursor	**1.85**	<0.001	−1.13	NS	1.30	NS	−1.06	NS
Immunoglobulin kappa light chain V	**1.82**	<0.05	−1.12	NS	1.06	NS	1.38	NS
Inter-alpha globulin inhibitor H2	**1.55**	<0.05	1.11	NS	**1.70**	<0.05	1.14	NS
Inter-alpha-trypsin inhibitor family heavy chain-related protein	**1.99**	<0.01	−1.03	NS	**1.52**	NS	−1.16	NS
Kininogen-1 isoform 2	**1.58**	<0.001	1.02	NS	1.07	NS	1.17	NS
Leucine-rich alpha-2-glycoprotein 1	**1.58**	<0.05	−1.11	NS	**1.82**	<0.05	−1.03	NS
Plasminogenisoform 1 precursor	**1.74**	<0.005	1.00	NS	1.12	NS	−1.19	NS
PREDICTED: similar to immunoglobulin lambda-like polypeptide 1	**2.57**	<0.01	−1.39	NS	1.21	NS	1.18	NS
Prothrombin; coagulation factor II	**1.96**	<0.001	1.08	NS	1.20	NS	1.06	NS
Putative uncharacterized protein DKFZp686G11190	**2.65**	<0.005	−1.33	NS	1.32	NS	1.26	NS
Recombinant IgG3 heavy chain	**2.59**	<0.05	−1.28	NS	1.36	NS	1.21	NS
Vitamin D-binding protein/group specific component	**2.37**	<0.001	−1.09	NS	1.37	NS	−1.07	NS

NS: p-value is not significant.

§ - Changes are relative increases or decreases from the second visit compared to the first expressed as averaged for each group.

The paucity of changes in the measured proteomes between visits in Groups 2 and 3 indicated that METH use had limited effect of these proteins at the time of the first visit, since METH abstinence in the context of recent use at the first visit (Group 2) or a history of METH use (Group 3) did not result in changes; as the amount of change in the proteome did not differ from that found in controls who were HIV negative and did not use METH (Group 4). However, many changes were found in Group 1, consisting of the significant increase of twenty one proteins, and a decrease of one protein. This effect of METH is very likely due to a recurring insult from taking METH. This can be either due to the continued use of METH over this period, and/or recent METH use; the positive urinary toxicology used to identify METH use indicates its use within the last three days.

**Figure 2:**
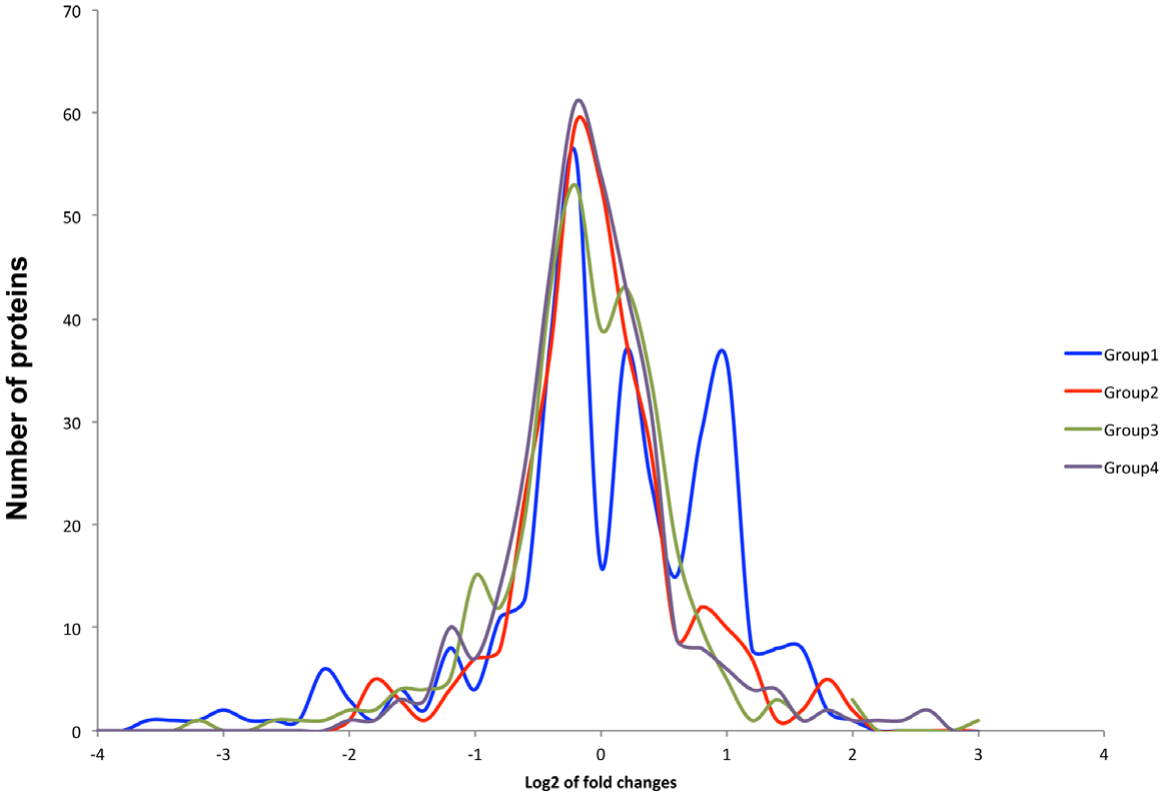
Distribution of the Log_2_ fold changes in proteins' expression in all groups. It is expected that proteins will be distributed around log2 of 0 ( = 1, no change between visits). We observed two additional peaks in Group 1 indicated by arrowheads; larger peak with log_2_ of 1 corresponding to 2-fold increase and smaller peak with log_2_ of approximately -2 corresponding to 7-fold decrease.

Figure 2 demonstrates that a specific subset of proteins, designated by the arrows, was differentially perturbed in HIV-infected persistent METH users (HIV+/persistent METH+). The majority of these proteins were increased in expression in association with persistent METH use. A smaller subset of proteins showed reduced expression. HIV positive individuals who discontinued METH use (Groups 2 and 3) showed profiles more similar to those of HIV negative METH negative subjects.

## Discussion

Use of METH is associated with multiple adverse health outcomes such as behavioral risks facilitating sexually transmitted diseases and mental health problems such as psychosis and depression [24], [25]. A strong association between METH use and increased risk of HIV has been substantiated by multiple studies [25], [26], [27]. Despite treatment, HIV infection is associated with a significant degree of neurocognitive impairment in one out of every seven subjects [28]. In SIV infected rhesus monkeys, METH treatment led to immune changes as well as increased virus in the brain [29]. Therefore, it can be expected that the combined effects of HIV infection and METH use will have not only additive or perhaps synergistic adverse effects on the brain, but also will have a detrimental impact on many physiological functions [30] including the immune system. We postulated that such effects would be reflected by changes in plasma proteome. Alterations in the plasma can affect the brain via communications between the periphery and the CNS, as well as through breach of blood-brain barrier (BBB) that can occur in HIV infection as well as drug abuse.

Interestingly the majority of significant changes were observed between visits in Group 1, those who did not discontinue using METH. Two major pathways were affected. First, was the complement cascade. This system is usually activated by HIV-1 infection alone; here continued METH use leads to further increase of C4A and C5 components. Such an increase likely represents non-specific response to an insult [31], [32]. The second activated system was blood coagulation and up-regulation of plasminogen, fibrinogen and kininogen. It has been shown that one toxic effect of METH use is disseminated intravascular coagulation (DIC, consumptive coagulopathy), which is a pathological activation of coagulation mechanisms [33]. As the small clots consume coagulation proteins and platelets, normal coagulation is disrupted; normal blood flow to organs is disrupted and this can lead to abnormal bleeding [34]. None of the subjects in this analysis had DIC but reported use of METH, at least in the setting of concurrent HIV infection, appears to result in less severe activation of coagulation pathways.

Increased expression of ceruloplasmin and hemopexin can represent a response to modulate increases in oxidative stress resulting from HIV infection and METH use. We expected that in response to METH exposure oxidative stress should be significantly enhanced and be correlated to increased expression of antioxidant enzymes, similar to our findings in SIV infected monkeys administered a chronic METH regimen [35]. Ceruloplasmin is a copper-binding glycoprotein oxidizing Fe^2+^ to Fe^3+^ ferroxidase without releasing radical oxygen species. Its main reported function is transport of iron across the cell membrane; however, several recent reports link this protein to an overall protective response of the host during increase of an oxidative stress. Ceruloplasmin is produced in the liver and our findings suggest that its expression increases during periods of METH use. These changes may remit following cessation of METH use, explaining why we did not observe changes in ceruloplasmin expression in patients after short or long term METH abstinence. In our previous plasma and cerebrospinal fluid (CSF) profiling experiments of HIV-infected individuals we also found ceruloplasmin to be differentially expressed [23], [36], [37]. Interestingly, this protein was down regulated in CSF of subjects with HIV dementia whereas it was up-regulated in plasma, suggesting that the CSF-to-plasma ratio of ceruloplasmin may be an important correlate of HIV-associated neurocognitive impairment.

The observation that changes in the plasma proteome were largely limited to subjects who continued to use METH was also unexpected. Because of this, we could not confidently identify a signature of METH use versus METH abstinence. Continued use of METH is by its nature an unstable condition, and users “crash” after METH binges [38]. Similarly the plasma proteome changes we found in Group 1 were not consistent with adaptive long-term changes, suggesting that METH use continues to lead to instability in normal physiology such as the complement and coagulation systems even during chronic use. It has to be noted that some of the subjects in Groups 2 and 3 were on treatment for HIV infection whereas none of those in Group 1 were treated for HIV. Our previous studies revealed that the proteome changes rapidly within the first two weeks of infection (acute phase) and comes back to background, especially if cART is implemented [22]. HIV-induced changes in proteome become obvious when viral infection is not well controlled and inflammation is on the rise. Therefore the relationship between METH use and HIV infection and the changes found in Group 1 is likely complex.

During the last decade of clinical and translational proteomics development much emphasis has been placed on technological development with expectations that more sensitive mass spectrometers and multi-level pre-fractionations will lead us to discovery of multiple new diagnostic biomarkers [39], [40]. However, the reality showed that although cataloging hundreds or even thousands of proteins in plasma and CSF has become more and more routine, quantitation and validation techniques are lagging [15], [41]. This makes it more difficult to draw conclusions about how to connect differentially expressed proteins to biology of disease and to judge whether a potential biomarker is specific and directly relevant to the pathology in question [42], [43]. Another layer of complexity is correlating genomic and proteomic data into one, synthesizing within a comprehensive and biologically meaningful scheme [44], [45]. Much progress has been made in this area during the last decade when genomic and proteomic data from cellular proteins are compared. In the case of secreted proteins, especially those expressed by multiple tissues, such connections are even harder to make [45], [46]. Also our knowledge about molecular mechanisms governing CNS and periphery communication is lagging and will require further investigations of BBB function to better interpret changes in proteomes of these two compartments. Considering studies performed so far [ref], there is no clear answer whether we should or should not expect such correlations and what they are indicative of biologically.

### Conclusions

Our study is the first report of systematic proteomic profiling of plasma samples, aiming to address the question of the effect of METH use or abstinence using well-defined groups of research subjects. Based on our results, we draw three conclusions for the effect METH use on the plasma proteome in the people living with HIV disease. First, changes in the hosts' responses to METH use may be short lived or require continued METH use to reach detectable levels. Second, the effect of METH is reflected by changes in plasma proteins that are linked to oxidative stress and inflammation. Third, METH use perturbs blood coagulation pathways, with upregulation of plasminogen, fibrinogen and kininogen. Consumption of clotting factors in a coagulation cascade may result in an imbalance between pro- and anti-coagulation. This could in turn increase the risk of both ischemic and hemorrhagic end-organ disease. Recent epidemiological studies demonstrated substantial increases in intracerebral hemorrhage and myocardial infarction in young adults abusing METH [47], thus our work may provide insight into such complications of METH use, whether in the setting of HIV infection or in its absence.
